# The truth is out there

**DOI:** 10.1186/2041-2223-4-24

**Published:** 2013-11-27

**Authors:** Mark A Jobling

**Affiliations:** 1 Department of Genetics, University of Leicester, University Road, Leicester LE1 7RH, UK

## 

Here’s a mysterious menagerie for you: the Omani owl, the hero shrew, Mondolfi’s four-eyed opossum, the Annamite striped rabbit, and the Khasian leaf-nosed bat. You’re unlikely to encounter even one of these curious critters, as they’re all rare and highly localised, and you’ll certainly never see them together in one zoological collection. What unites them is that they are all ‘new species’ , discovered and named in the last few years. None have made headline news, it’s true, but certainly more of a splash than the enormous supporting cast of novel beetles, arriving in an unending procession, but sadly ignored by all but the most dedicated entomologist.

Despite the thoroughness with which our own species already seems to have scoured the planet, new kinds of non-human inhabitants turn up all the time, thanks to intrepid zoological explorers and taxonomists, and aided by DNA-based analysis to demonstrate their positions in the tree of life. Such work is laborious, but luckily there’s another simpler way to find a new species – split an existing one. For example, twenty years ago there was only one species of orangutan, with Sumatran and Bornean populations designated subspecies; now these have official species status. Such decisions can have important and beneficial impacts on conservation efforts, particularly when, as with the unfortunate orangutans, or other newly discovered primates such as the Arunachal macaque, Caqueta titi monkey and Highland mangabey, they are highly endangered by habitat destruction and hunting.

As if all this wonderful real life weren’t enough, some people have a need to believe in some less tangible things. Setting aside the clearly supernatural, from angels to zombies via fairies and trolls, there is a host of hypothetical animals that have been claimed to lurk out there – the creatures of cryptozoology. Encyclopaedist George Eberhart sets down some criteria for what constitutes a ‘cryptid’ [1], and classifies them into groups. First, some threshold of significance needs to be met – they must be “big, weird, dangerous, or significant to humans in some way”, and this therefore excludes the humble beetle. Also, some controversy needs to attach to their status, such as vociferous claims for their existence, combined with equally vociferous claims that they are imaginary. Among Eberhart’s cryptid categories are distribution anomalies, in which a known species is reported well outside its normal range; these include British sightings of panthers [2]. More interesting are the alleged survivals of extinct species, including contemporary reports of the proverbially dead dodo, and the famous Loch Ness Monster, claimed by some to be a plesiosaur species that miraculously survived the Cretaceous–Tertiary extinction event, 66 million years ago. Loch Ness watchers have produced a series of blurred black and white photographs that are claimed either to be the monster, or less exciting things, including the wake of a boat, a dog swimming towards the camera carrying a stick, and a model dinosaur head attached to a toy submarine. This raises the difficult issue of hoaxes – the North American legend of the jackalope, a jack-rabbit with antlers, led many creative taxidermists to get to work.

Among cryptids that have no parallel in the fossil record or in living species are a number of humanoid creatures that perhaps lurk in the woods and mountains; these include the American bigfoot (also known as sasquatch), the Himalayan yeti, and the Australian yowie. Stories abound, and in the case of bigfoot are bolstered by photos of suitably large footprints, and grainy images of a creature that some infidels insist is a man in a gorilla suit. A video (available on YouTube) shows either a dozing bigfoot, or a person asleep under a rug, depending who you ask.

Late last year a scientific paper appeared [3] claiming DNA-based analysis of bigfoot specimens, including whole-genome sequences. Don’t bother searching for it in PubMed, though, because it was published as the first and only paper in an online journal called *De Novo*[4]. Initially, the paper was sent to *Nature*, and also the *Journal of Advanced Zoological Exploration in Zoology*, which appears to be another pop-up journal [5]. If you are interested in the details, you can read the paper itself, plus reviewers’ comments and the authors’ responses [6], and make up your own mind about the robustness or otherwise of their claims. The methods used sound good, including sequencing of the entirety of the maternally-inherited mitochondrial DNA (mtDNA), and even the use of next-generation sequencing to analyse whole genomes in three cases. The DNA sources are 113 assorted samples of hair, blood, mucus, toenail, bark scrapings, saliva and skin with hair and subcutaneous tissue attached, contributed by a large number of bigfoot enthusiasts.

And what of the data? Mitochondrial analysis showed only human sequences, belonging to a number of types (haplogroups) typical of the modern US population. Other sequencing indicated that “the species possesses a novel mosaic pattern of nuclear DNA comprising novel sequences that are related to primates interspersed with sequences that are closely homologous to humans.” However, there’s no attempt to focus on these ‘novel sequences’ and figure out what they might be. On the basis of a confusing mass of data and a certain amount of evolutionary naivety, the authors conclude that “the data conclusively proves that the sasquatch exist as an extant hominin and are a direct maternal descendent (sic) of modern humans.” It would be exciting if this were true, but unfortunately, it seems more likely that the samples are a mix of modern human DNA and some animal DNA or other.

The United States is a populous and technologically advanced place, home to over 327 million mobile phones, so it’s puzzling that the bigfoot photos continue to be of such poor quality. And where do these creatures go when they die? A properly examined corpse would go a long way to silencing the sceptics. The Himalayas, on the other hand, are remote and relatively unpopulated, so it seems possible that yetis may actually live (and die) there undetected. Enter Bryan Sykes, himself something of a semi-mythical hominin in the scientific community since his retirement from the hurly-burly of standard research endeavour almost a decade ago. He has been in the media over the last few weeks describing his yeti project – like the bigfoot folks, he apparently sourced alleged yeti samples from anyone who was interested in contributing them. This is a worry, particularly given the possibility of mischievous hoaxers. Mitochondrial sequences apparently resemble those found in 120,000-year-old polar bear samples from Svalbard [7], leading to the suggestion that yetis are some kind of long-surviving bear species. This seems plausible, but it’s not yet published – Sykes says it will be, but hopefully not in *De Novo*.
